# Microbial Diversity Profiling of Gut Microbiota of *Macropus giganteus* Using Three Hypervariable Regions of the Bacterial 16S rRNA

**DOI:** 10.3390/microorganisms9081721

**Published:** 2021-08-12

**Authors:** Christian O’Dea, Roger Huerlimann, Nicole Masters, Anna Kuballa, Cameron Veal, Paul Fisher, Helen Stratton, Mohammad Katouli

**Affiliations:** 1 Genecology Research Centre, School of Health and Sport Sciences, University of the Sunshine Coast, Maroochydore, QLD 4558, Australia; christiancodea@gmail.com (C.O.); NMasters@USC.EDU.AU (N.M.); AKuballa@USC.EDU.AU (A.K.); 2 Marine Climate Change Unit, Okinawa Institute of Science and Technology (OIST), 1919-1 Tancha, Onna-son, Okinawa 904-0495, Japan; roger.huerlimann@jcu.edu.au; 3 Seqwater, 117 Brisbane Street, Ipswich, QLD 4305, Australia; Cameron.Veal@seqwater.com.au (C.V.); Paul.Fisher@seqwater.com.au (P.F.); 4 School of Environment and Science, Griffith University, Nathan, QLD 4111, Australia; h.stratton@griffith.edu.au

**Keywords:** gut microbiota, diversity profiling, macropods, 16S rRNA

## Abstract

Animal faecal contamination of surface waters poses a human health risk, as they may contain pathogenic bacteria or viruses. Of the numerous animal species residing along surface waterways in Australia, macropod species are a top contributor to wild animals’ faecal pollution load. We characterised the gut microbiota of 30 native Australian Eastern Grey Kangaroos from six geographical regions (five kangaroos from each region) within South East Queensland in order to establish their bacterial diversity and identify potential novel species-specific bacteria for the rapid detection of faecal contamination of surface waters by these animals. Using three hypervariable regions (HVRs) of the 16S rRNA gene (i.e., V1–V3, V3–V4, and V5–V6), for their effectiveness in delineating the gut microbial diversity, faecal samples from each region were pooled and microbial genomic DNA was extracted, sequenced, and analysed. Results indicated that V1-V3 yielded a higher taxa richness due to its larger target region (~480 bp); however, higher levels of unassigned taxa were observed using the V1-V3 region. In contrast, the V3–V4 HVR (~569 bp) attained a higher likelihood of a taxonomic hit identity to the bacterial species level, with a 5-fold decrease in unassigned taxa. There were distinct dissimilarities in beta diversity between the regions, with the V1-V3 region displaying the highest number of unique taxa (*n* = 42), followed by V3–V4 (*n* = 11) and V5–V6 (*n* = 8). Variations in the gut microbial diversity profiles of kangaroos from different regions were also observed, which indicates that environmental factors may impact the microbial development and, thus, the composition of the gut microbiome of these animals.

## 1. Introduction

Faecal contamination of environmental waterways from warm-blooded animals is one of the leading causes of surface water degradation and risk to human health through a proliferation of zoonotic pathogens [1]. Understanding these animals’ possible faecal contribution is vital for water quality and catchments management from a Water Industry perspective. Wild, agricultural, and domestic animals may contaminate waterways through direct defecation into surface waters. Through transport in surface runoff during rainfall events [2,3], wild animals, who commensally exist alongside agricultural animals in open farmland acreages, contribute to the microbial load of adjacent surface waters [4]. Kangaroos (*Macropus* spp.) are considered to be the most prevalent Australian wild animal, with their population estimated to comprise between 25–50 million individuals across Australia [4]. Kangaroos commonly occur in regions that are inhabited and used by livestock and humans, and which include surface waters that are used as a drinking water source. As a ruminant animal, the kangaroo has been identified as hosting several human pathogens within their gut microbiota, with the potential to cause zoonotic harm to humans in rare cases [5,6]

Due to the diversity of gut microbiota and complex bacterial signatures between animals and humans, microbial source tracking (MST) methods have been developed to identify bacterial taxa that may be novel in order to distinguish animals that contribute to freshwater faecal pollution [7]. As animal faecal contamination is less distinguishable as a point source, identifying the faecal pollution’s source once it enters the waterway is difficult [8]. Recent advances in MST methods, such as high-throughput sequencing (HTS) technologies, have allowed us to discover novel microorganisms associated with the host species by characterising the gut microbial populations of humans and animals [8,9]. This technology has dramatically improved the taxonomic classification of prokaryotes, mainly through the use of the ~1500 bp, 16S rRNA gene of the bacterial ribosome. While utilising the full-length 16S rRNA gene to characterise bacterial communities is considered to be the gold standard for the full taxonomic resolution of any sample, using one of the nine hypervariable regions (HVR) of the 16S rRNA gene allows for more cost-effective analyses [10,11,12]. However, limitations surround this HTS method, as there is no single region of the 16S gene that has been identified to date that can accurately represent its entire length or mimic its resolving power [10,13]. This has led to further research into the taxonomic classification method utilising other validation methods [14,15,16,17].

Before HTS, most researchers used Sanger sequencing to characterise bacterial communities, a process which often required time-consuming methods, such as cloning or bacterial isolation, before sequencing [18]. However, Sanger sequencing also allowed for “Meta” analyses of a sample, allowing for longer reads which were often greater than 500 base pairs. [19]. The increased speed, reduced cost, simpler protocols, and comparable read lengths of HTS have allowed this technology to supersede Sanger sequencing [20]. Regardless of the sequencing method, the results are represented in operational taxonomic units (OTUs), which usually identify an organism to the genus or species level [21,22].

The nine HVRs of the 16S rRNA gene are within conserved regions, facilitating primer design and PCR amplification of the target sequences [23]. Previous studies have utilised only one or two of these regions due to shorter read lengths becoming more manageable than the total length ~1500 bp gene [24]. Conversely, a larger amplicon size will reduce misinterpretation of taxa [14,17,25,26,27,28]. These variables can make the selection of the appropriate HVRs troublesome to navigate. Additional considerations include accuracy in the sequencing platform analysis, filtering of data, and denoising the raw data using bioinformatics pipeline software, affecting the overall bacterial community and diversity outcome [29]. Although Australian macropods, including the Eastern Grey Kangaroo, are common to the South East Queensland (SEQ) region of Australia, there is very little information about the bacterial diversity of these animals’ gut microbiota [30]. We postulated that selecting an appropriate HVR of the 16S rRNA gene for diversity profiling of gut microbiota would provide the discriminatory power to identify not only the same resolution as the full 16S gene, but also the unique bacterial signatures that can be used to identify the macropods’ source of faecal pollution in waterways.

This study was undertaken in order to evaluate the potential of three popular HVRs of the 16S rRNA gene (V1–V3, V3–V4 and V5–V6) for detecting the phylogenetic diversity, species richness, and beta diversity of gut microbiota of Eastern Grey Kangaroos from six regions within SEQ, Australia. Furthermore, the study’s purpose was to isolate and identify unique molecular genetic targets in the gut bacterial microbiome of the Eastern Kangaroo to be ultimately used in tracking their movement and faecal contamination in recreational water sources.

## 2. Materials and Methods

### 2.1. Collection of Faecal Samples

**Figure 1:**
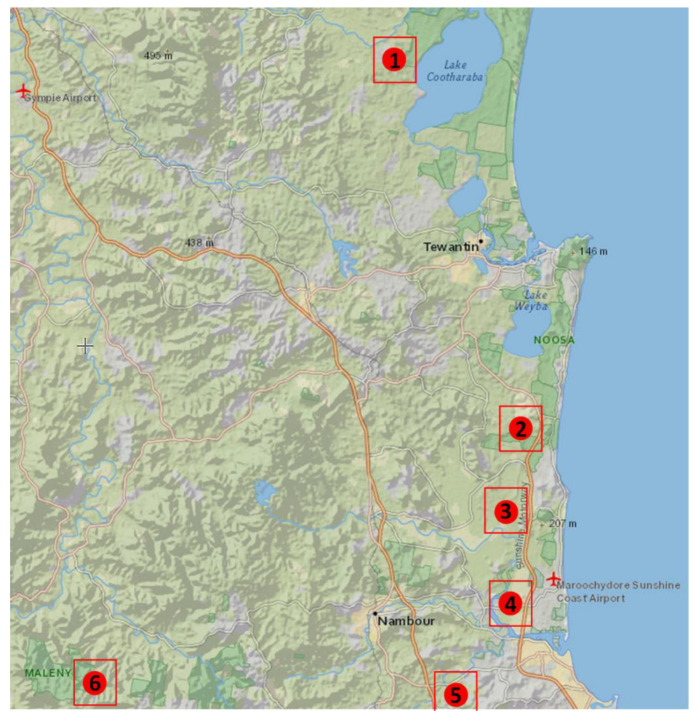
Map of Eastern Grey Kangaroo mob sites where faecal samples were collected in the southeast of Queensland, Australia. Sites in order: Cootharaba, Elanda Point, Yaroomba, Twin Waters, University of the Sunshine Coast, Maleny National Park.

Thirty individual faecal samples were collected from known Eastern Grey Kangaroo mob sites across SEQ, Australia (Figure 1). All sites were home to discrete mobs, with no recently known mixing of populations. Sites included Elanda Point (site 1), Sippy Downs (site 2), Yaroomba (site 3), Maleny National Park (site 4), Cootharaba (site 5), and Twin Waters (site 6) (Figure 1). Where possible, fresh faecal samples were collected either after observing an individual animal defecating or from grounds where the animals were previously observed. Faecal samples were collected using sterile, wide-mouth sample containers and transported to the laboratory on ice for subsequent gDNA extraction within six hours of sampling. The kangaroos from which the faecal samples were collected were not observed to be of ill health at the time of sampling. Property owners where the kangaroos reside were also questioned regarding their observations of the mob’s general health. Faecal scats (>10 g) were taken from the innermost section of the faecal samples; this internal portion of the scat was used in order to minimise external contaminants.

### 2.2. DNA Extraction of Faecal Samples

In accordance with the manufacturer’s protocol, DNA extraction of individual faecal samples was carried out using the Qiagen QIAamp Power Faecal DNA kit (Qiagen, Valencia, CA, USA). The elution of DNA was modified using 150 µL of C6 buffer instead of the 100 µL that is described in the protocol. The yield and purity of the genomic DNA were confirmed using NanoDrop Microvolume Quantitation (Thermo Scientific, Waltham, MA, USA) and gel electrophoresis. Extracted DNA samples were stored at −20 °C until further analysis could be performed. Extracted DNA from five individual faecal samples was pooled post-extraction according to the region where the sample was initially collected. An equal concentration (20 ug/µL) of each faecal sample was used to create the final pooled sample to ensure equal representation and minimise bias. The pooled gDNA samples were submitted to the University of Minnesota Genomics Centre (UMGC), USA, for 16S rRNA amplicon sequencing of the V5–V6 region, while regions V1–V3 and V3–V4 were sequenced at the Australian Genome Research facility (AGRF), Melbourne, Australia.

### 2.3. 16S rRNA Amplification and Miseq Sequencing

Amplicon sequencing of the HVRs was sequenced utilising the Illumina MiSeq platform (San Diego, CA, USA) with two × 300 base pairs paired-end chemistry. Sequencing and library preparation was undertaken with the Australian Genome Research Facility, Brisbane, Australia and was performed in real-time using the MiSeq Control Software (MCS) v2.6.2.1 and Real-Time Analysis (RTA) v1.18.54, MiSeq instrument computer. Sequencing primers and reagents are described elsewhere [31] (Table 1). Resultant reads were subjected to quality control assessment ‘FASTQC’ (V3) and trimmed using the ‘Trimmomatic’ adaptor trimming pre-processing tool (V0.39) [32]. The source libraries, sequencing primers, reagents, equipment, and pipelines were conducted as mentioned previously [33,34]. Primer sets used to amplify the HVRs of the 16S rRNA gene are shown in Table 2. KAPA HiFidelity Hot Start Polymerase was used for PCR amplification that utilised the following cycling conditions: 95 °C for 5 min, followed by 25 cycles at 98 °C for 20 s, 55 °C for 15 s, and 72 °C for 1 min. Illumina adapter and barcode sequences were added with an additional ten cycles of PCR using the dual index method [35]. Amplicons were gel purified, pooled, and paired-end sequenced at a read length of 300 nt on the Illumina MiSeq platform (Illumnia, Inc., San Diego, CA, USA). Region V5–V6 Amplicon sequencing was performed by the University of Minnesota Genomics Centre (http://genomics.umn.edu/ accessed 15 June 2018) using standard workflows and the Q.C. program as described above [7].

### 2.4. Bioinformatic Analysis of Data

The processing of raw sequence 16S rRNA data used Quantitative Insights into Microbial Ecology (QIIME2), an open source, a metagenomic bioinformatic pipeline analysis tool [39]. This bioinformatic methodology was also mentioned elsewhere [40]. Samples were indexed into categories that aligned with a metadata file. Samples were first subjected to joining forward and reverse reads using artefact ‘Paired-End Sequences with Quality’ [39]. The joined reads were denoised and filtered of reads compromising the final output. The ‘DADA2 denoise-paired’ artefact trimmed forward and reverse primer ends of the amplicons and truncated the length of the region from the sequence reads [39]. Utilising a denoising algorithm also removed error in PCR recombination (chimeras), and a sequencing identity algorithm that removed reads with less than a 97% similarity cutoff. The resultant output QZA files underwent a ‘Feature table Merge’ of the denoised amplicon data to be analysed downstream. Sequence alignment, mask/filter alignment, and production of phylogenetic tree files were created. Biases were removed by ‘alpha rarefaction’ in order to accommodate and filter only quality reads at their maximum read length depths. Finally, the taxonomic assignment was undertaken with an open reference database, ‘SILVA-132-99 classifier’, for its regularly updated, quality-controlled resource data [41]. Phylogenetic diversity profiling was carried out in order to determine relative abundance -of bacterial genera between populations in different samples.

Exported taxonomy files were converted to the BIOM format for subsequent output analysis into the open-source metagenomic data facilitation software CALYPSO [42]. Data Filtering removed samples with short sequence read counts (<1000 sequence reads) and OTUs with low abundance (<1%). Furthermore, only the top 3000 taxa were included and filtered by mean. Data were normalised by dividing the number of sequence reads by the total number of reads within each sample by total sum normalisation (TSS).

Host-specific microbial richness was estimated using Alpha diversity matrices Chao1, ACE, Shannon’s diversity, and Simpson’s diversity indices. Distance relationships between microbial community groups were performed using Bray-Curtis [43]. Distance matrices by beta diversity measurements were visualised by Principle Coordinate Analysis (PCoA) using CALYPSO [42]. Unique taxa and incidence mapping comparing each of the three regions tested were calculated by group analysis at the genus level in order to identify unique and shared taxa between HRVs [42,44].

### 2.5. Statistical Analyses

Statistical analysis was performed using Graph Pad Prism software version 9.01 for Windows (GraphPad Software, San Diego, CA, USA). Differences between the bacterial richness obtained from the three HVRs were determined using the Kruskal-Wallis analysis of variance (ANOVA) and the values were considered statistically significant where *p* < 0.05.

## 3. Results

### 3.1. Sequence Data Summary and Depth

**Figure 2:**
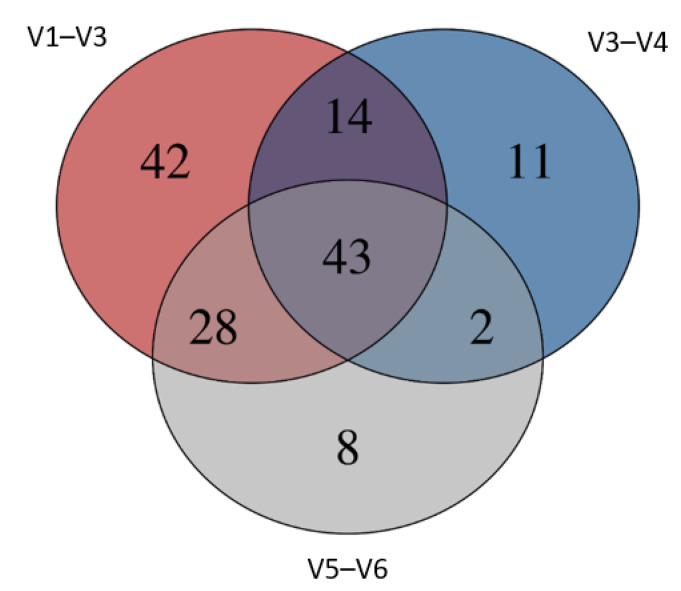
Group visualisation of taxa abundances across three chosen hypervariable regions of the 16S rRNA gene. The top 300 most abundant genera were utilised for visualisation of shared and exclusive genera.

Illumina sequencing of each of the three HVRs resulted in: 1,962,938 demultiplexed sequence reads for the V1–V3 region; 997,779 sequence reads for region V3–V4; and 271,184 reads for region V5–V6. Post rarefication filtering to 10,000 reads per sample was employed before alpha diversity metrics were analysed. Chao1 and ACE bacterial richness indices showed a significantly higher bacterial richness in region V1–V3 out of all three regions (*p* < 0.05) (Figure 2). Shannon’s and Simpson’s diversity indices displayed a significantly broader diversity within the V1–V3 region (Figure 2).

### 3.2. Taxonomic Composition by Hypervariable Region

The most abundant OTUs within the three HVRs of the 16S rRNA gene were identified as belonging to phyla Proteobacteria, Firmicutes and Bacteroidetes, which comprised up to 91% of the total bacterial coverage (Table 2). Proteobacteria was the predominant phyla in HVR V1–V3 (49.7%) and Firmicutes was predominant in HVR V3–V4 (47.4%) and V5–V6 (45.1%). Bacteroides, a known contributor to the upper portion of all mammalian’s gastrointestinal tracts [45], was also present as the third most abundant bacterial phyla within all three HVRs tested (Table 2).

**Figure 3:**
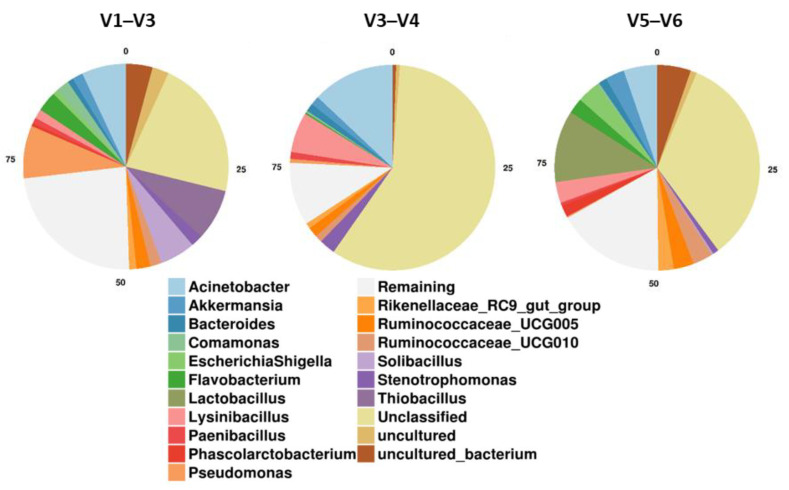
Microbial community composition at the genus level, within the three hypervariable regions of the 16S rRNA gene.

The highest proportion of unclassified bacterial OTUs was observed in HVR V3–V4 (58.5%). In comparison, HVRs V1–V3 and V5–V6 displayed much lower unclassified OTUs, 21.7% and 33.2%, respectively (Figure 3).

**Figure 4:**
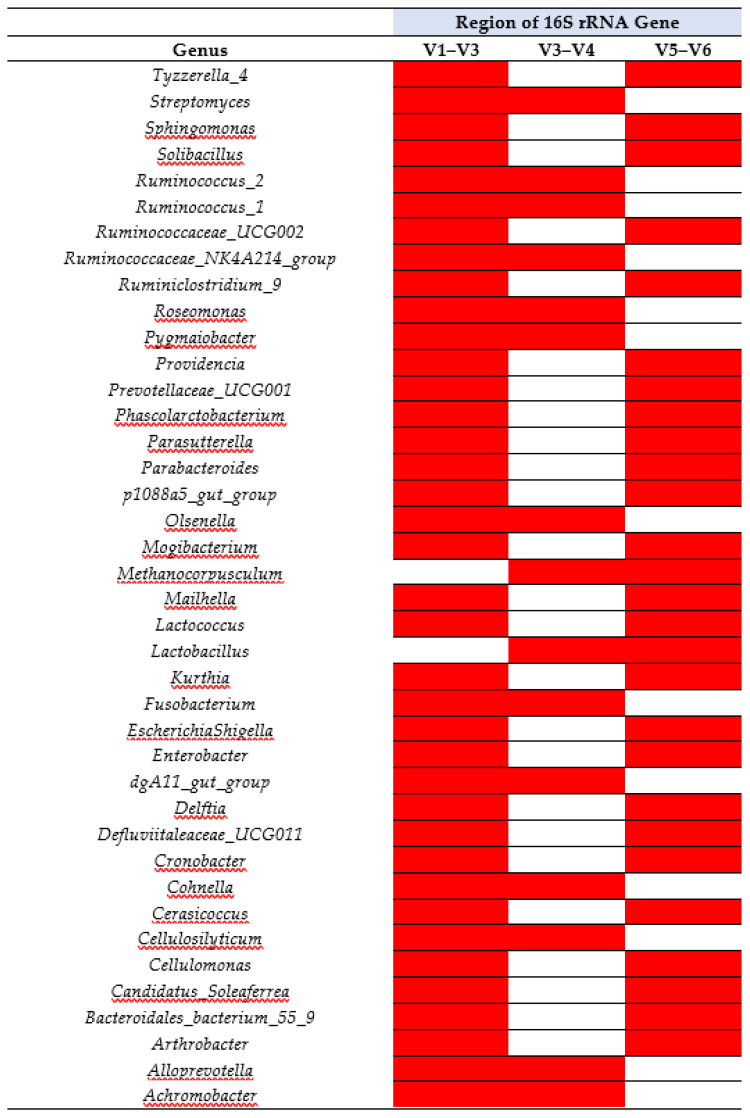
Incidence map displaying bacteria found at the genus level in each of the three hypervariable regions of the 16S rRNA gene (V1–V3, V3–V4 and V5–V6). Presence (red), and absence (white).

The top 40 genera underwent incidence analysis to determine if all three regions were able to detect any of the given genera. Of the top 40 genera, 95% were sensitive to being detected by two out of the three HVRs (Figure 4).

### 3.3. Taxonomic Composition by Geospatial Region

The taxonomic composition of the faecal microbiota of Kangaroos from the six regions was compared using the results obtained from each of the three HVRs.

**Figure 5:**
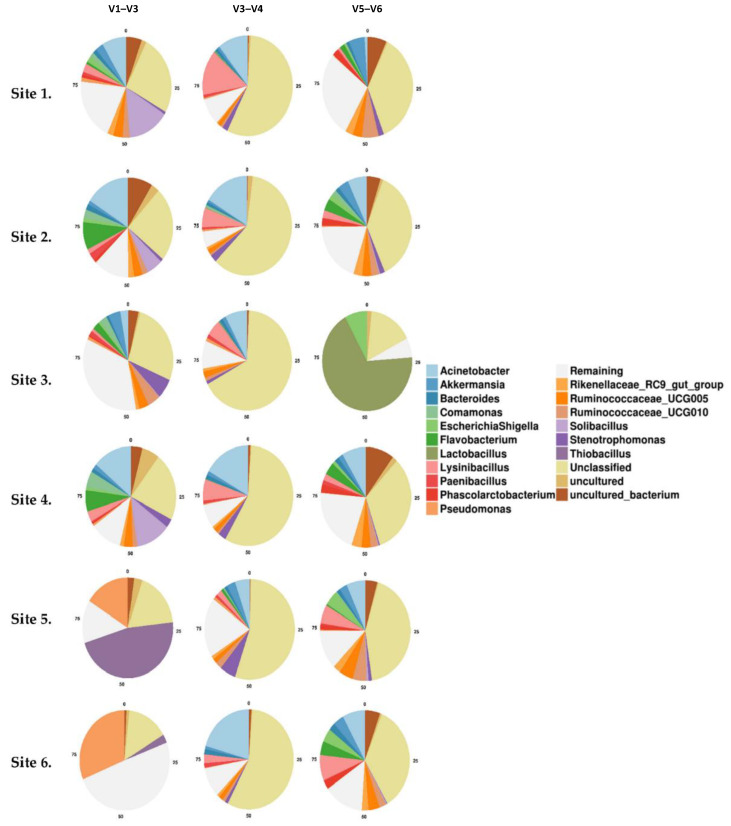
Quantitative visualisation of bacterial phyla taken from kangaroo faecal material at six sample sites within South East Queensland. Samples were tested against each of the three hypervariable regions of the 16S rRNA gene, V1–V3, V3–V4, and V5–V6. Sites are displayed in order: 1—Cootharaba, 2—Elanda Point, 3—Yaroomba, 4—Twin Waters, 5—the University of the Sunshine Coast, 6—Maleny National Park.

Bacterial OTU abundance was found to vary according to geographical location. Firmicutes were the predominant phyla within sites 5 (Cootharaba), 1 (Elanda Point), 6 (Twin Waters), and 2 (Yaroomba) for all three HVRs. However, V1–V3 analysis identified Proteobacteria as the most abundant at sites 4 (Maleny National Park) and 2 (Sippy Downs) (89.6% and 85.6%, respectively) (Figure 5).

### 3.4. Group-Specific Bacterial Communities

Unique taxon differences between HVRs used in this study were conducted using the permutational multivariate analysis of variance (Figure 2). The V1–V3 HRV of the 16S gene showed the highest level of unique taxa identified among the three regions (*n* = 42). The least unique genera were identified in HVR V5–V6, while all three HVRs shared a total of 43 genera (Figure 2).

### 3.5. Beta Diversity

**Figure 6:**
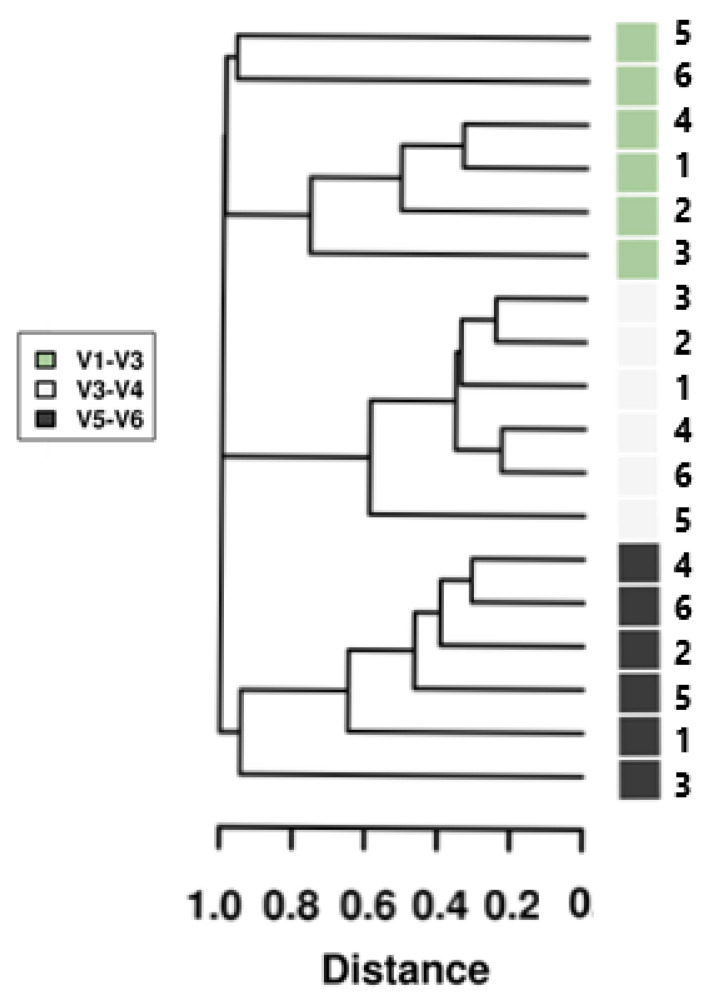
Hierarchical dendrogram displaying Bray-Curtis distances from samples of Eastern Grey Kangaroo faecal gDNA. Samples were analysed against the 16S rRNA bacterial gene regions V1–V3, V3–V4 and V5–V6. Sites are displayed in order: 1—Cootharaba, 2—Elanda Point, 3—Yaroomba, 4—Twin Waters, 5—the University of the Sunshine Coast, 6—Maleny National Park.

Beta diversity profiling identified dissimilarities among bacterial communities within all three HVRs tested and across geographical sampling sites for the Eastern Grey Kangaroo populations. PCoA analysis revealed the V3–V4 region as the least differentiating diversity clustering of all the HVRs tested. Results indicate the most significant geographical diversity was observed between the ‘University of the Sunshine Coast’ (USC) & ‘Maleny National Park’ (MNP) sampling sites, when analysing HVR V1–V3. Similarity coefficients between the composition of the six pooled gut microbiota of EGK using three HVRs showed a higher similarity among the pooled gut microbiota of Kangaroos within each HVR analysis than between HVRs (Figure 6). Sample sites were also analysed for similarity; however, no significant similarity was observed between the six sample sites (Figure 6).

For the first time, this research presents insights into the effectiveness of the 16S rRNA gene sequence analysis to reconstruct phylogenies using three of the nine favoured, highly conserved regions of the 16S rRNA gene from macropod species *Macropus giganteus*; The Eastern Grey Kangaroo. The results of this experiment suggest that the bacterial phylogenies obtained from the testing of kangaroo gut-faecal material vary depending on which HRV of the 16S rRNA is analysed. Currently, no single HVR (neither in the literature nor from our analysis) can be defined as a standard. Prior research of this high-throughput sequencing technology using various HRVs described certain regions as adept at distinguishing bacterial OTUs over others [46]. Our research aligns with other analyses that suggest that short read HRV’s are not as effective in achieving the full taxonomic resolution of the full length 16S gene (~1500 bp) [13]. Analysis of gut microbiota and the diversity of bacteria in faeces may be affected by, but not limited to, several factors, which include (a) gDNA extraction method applied to faecal samples, (b) raw data processing, and (c) sequencing depth and microbiota improvement post-low-frequency sequence removal. Previous studies show that the efficacy of bacterial phylogenetic derivation may be compromised if the DNA extracted from animal faecal samples contains ethylenediamine-tetra acetic acid (EDTA) [46]. The use of EDTA has also increased the abundance of phylogenies such as Proteobacteria and decreased the abundance of Firmicutes [46]. Results from our study indicate that Proteobacteria are the predominant bacterial phyla when analysed using HVR V1–V3, while V3–V4 and V5–V6 predominantly identified Firmicutes. This phylogenic shift in the most abundant bacterial communities is also documented in earlier research [47].

Illumina sequencing of the three HVRs in our study resulted in region V1–V3 displaying a 1.96-fold increase in the sequence read count over region V3–V4 and a seven-fold increase over region V5–V6. We also found that removing low frequency reads in-silico aids in a higher sensitivity of bacterial diversity. This removal of singletons decreases overall error rates, as also described elsewhere [29,48,49]. A loss of ~90% of the original reads post denoising protocol (DADA2) was observed for HVR V1–V3. This degree of denoising is a common trait for HVR V1–V3 when undertaking this method, as the original reads are removed from the analysis due to a failure to merge the paired reads. We used the forward reads only, as a higher yield in reading outputs can be attained in analysing our data. However, since single read runs are faster and cheaper, future sequencing projects using this primer set could use single-end sequencing.

It is acknowledged that the full length of the 16S rRNA gene is more appropriate in achieving a more definitive resolution of the taxa within a sample [50]. Identification of errors in amplicon sequencing are attended to by utilising quality filtering bioinformatic pipeline scripts [39,51,52]. There exists multiple denoising (error correcting) bioinformatic pipelines for resolving amplicon sequencing variants. By using the bioinformatic post denoising protocol (DADA2), the identification of PCR chimeras and correcting of sequence amplicon errors assists in decreasing the resultant unclassified taxa of up to a 97% similarity cut-off. This aids in a more discriminatory OTU identification [13,53,54].

The predominant bacterial phylogeny within the V1–V3 region was Proteobacteria. The other two HVRs tested were comprised of communities of Firmicutes as the dominant phyla. Research published previously indicates a high presence of Proteobacteria, evaluating the HVR’s ability to quickly identify the bacterial sequences during high-throughput sequencing in faecal microbiome samples [47,55]. There exists no gold standard for utilising the correct HVR for the target bacterial community of interest. The sequencing of multiple regions exists in modern HTS research for a more comprehensive analysis of the sample of interest [50].

The literature suggests that the HVR preference in modern HTS depends on the microbiome source being tested. Analysis of alpha and beta diversity in our study showed that the V1–V3 region of the 16S rRNA gene held the most significant species richness when tested for alpha diversity. Conversely, beta diversity results displayed dissimilarity in the region holding the highest bacterial species between all three HVRs. A greater level of species richness can translate to a community composition that is not fundamentally similar.

Pitfalls relating to HTS technology pertain to the reproducibility of results using this technology. Sample processing and the extraction stage are related to the specificity of the bacteria present post metagenomic analysis. PCR based confirmation steps would hamper the effectiveness and affordability of attempts to confirm HTS analysis accuracy [56]. Measures to prevent this could include multiplex PCR which would enable the observable phyla within a DNA sample to help confirm HTS analysis [57].

This study hopes to show which HRV can be used as a comparative analysis in substitution of the full length ~1500 bp 16S rRNA gene. Through previous HRV comparison studies, it is evident that the conditions of a sample and the region of the 16S rRNA greatly dictate the microbial diversity of a sample. The literature is largely comprised of research into region comparisons across samples in isolation. It is noticed that through comparison of taxonomic percentages, not all HRVs display a consistent detection of genera across the 3 regions in this study, even if an equimolar mix of samples were used on the same HRV primer set [15,58]. Additionally, associated environmental factors such as diet, health and age can impact an animals bacterial microbiome [59].

It is not currently feasible to creating a database of suitable HRVs to use on a specific sample, in this case the gut microbiome of an animal species. Variability of any sample used against any HRV has the potential to differ in sequence variation, no matter how uniform the samples may be.

## 4. Conclusions

In conclusion we revealed that the gut microbiota of kangaroos examined in this study was diverse. However, we could not identify unique taxa as potential kangaroo specific faecal markers that could be used as a tool for identifying faecal contamination of surface waters by macropods. Our results did suggest that, depending on which HVR of the 16S rRNA is used, significant differences in bacterial phylogeny will be obtained, making it difficult to suggest which bacteria taxa could be nominated for further investigation in order to pinpoint a candidate for MST purposes. Secondary confirmation methods such as quantitative PCR and alternative bioinformatic pipeline methods to discover variances in data could provide a better picture of the bacterial markers specific to these animals. Nonetheless, we found a combined use of three variable regions of 16S rRNA could substitute for the use of the entire ~1500 bp gene (the full-length gene). Although this was a more costly approach to bacterial phylogenetic testing, it is more suitable for a better understanding of the gut microbial diversity and the unique taxa of macropods. Future studies are needed to investigate direct comparison of the three HVRs used with samples analysed along the full length 16s gene.

## Figures and Tables

**Table 1:** Primers sequences used in this study for amplification of three hypervariable regions.

Region Name	Primer Name	Primer Sequence	Length	Reference
V1–V3	27F	AGAGTTTGATCMTGGCTCAG	480 bp	[36]
	519R	GWATTACCGCGGCKGCTG		
V3–V4	341F	CCTAYGGGRBGCASCAG	569 bp	[37]
	806R	GGACTACNNGGGTATCTAAT		
V5–V6	784F	RGGATTAGATACCC	280 bp	[7,38]
	1064R	CGACRRCCATGCANCACCT		

**Table 2:** Taxonomic composition of the most abundant bacterial phyla when tested for three HVRs (V1–V3, V3–V4, and V5–V6) of the 16S rRNA genes.

Taxa (Phylum)	V1–V3 Mean	Taxa (Phylum)	V3–V4 Mean	Taxa (Phylum)	V5–V6 Mean
Proteobacteria	49.65%	Firmicutes	47.39%	Firmicutes	45.14%
Firmicutes	28.07%	Proteobacteria	29.25%	Proteobacteria	19.55%
Bacteroidetes	12.02%	Bacteroidetes	14.50%	Bacteroidetes	18.18%

## Data Availability

All data are contained within the manuscript.
